# Wound Lavage in Studies on Vital Pulp Therapy of Permanent Teeth with Carious Exposures: A Qualitative Systematic Review

**DOI:** 10.3390/jcm9040984

**Published:** 2020-04-01

**Authors:** Amina Munir, Matthias Zehnder, Dan-Krister Rechenberg

**Affiliations:** Preventive Dentistry, Periodontology and Cariology, Center for Dental Medicine, University of Zurich, 8032 Zurich, Switzerland; amina.munir@zzm.uzh.ch (A.M.); matthias.zehnder@zzm.uzh.ch (M.Z.)

**Keywords:** vital pulp therapy, pulp wound, sodium hypochlorite, disinfection, debridement, pulp survival

## Abstract

The aim of this study was to systematically review pulp wound lavage in vital pulp therapy (VPT). A search was conducted in six life science databases to identify clinical trials carried out on permanent teeth with a carious pulp exposure and a recall interval of at least six months. Twenty-seven trials of low to moderate risk of bias (RoB-2 and ROBINS-I) were included. Data was extracted and analyzed regarding study characteristics and methods used for pulp wound lavage. The agent used for pulp wound lavage was specified in all included trials. Most of the identified trials (23/27) randomized the pulp capping material. Many (14/27) reported the use of sodium hypochlorite (NaOCl); ten used only saline or water. One trial was identified that compared pulp wound lavage with 2.5% (NaOCl) to saline, another compared 5% glutaraldehyde to water, both in immature molar pulpotomies. Both studies were underpowered. Neither showed a significant difference between treatments. The use of NaOCl was positively correlated to recent year of publication and use of hydraulic calcium silicate cements for pulp capping (*p* < 0.05). In conclusion, despite a lack of well-designed trials on pulp wound lavage in VPT, a trend towards using NaOCl for this purpose was observed.

## 1. Introduction

Vital pulp therapy (VPT) is the treatment of the exposed pulp of a tooth with the aim of keeping it healthy and free from infection. This is done in teeth that have been damaged by caries, trauma or restorative procedures [1,2]. The different types of VPT can be categorized into indirect and direct pulp capping, partial pulpotomy and full (coronal or pulp chamber) pulpotomy. This is done according to the extent of pulp exposure, perceived stage of bacterial infiltration and damage to the dental pulp [3].

It has been shown that as soon as caries infiltrates the enamel an initial reaction of the adjacent pulp tissue can be seen histologically [4]. However, if the softened and carious dentine is excavated completely without exposure of the pulp, healing is usually possible [5]. In cases with pulp exposure, pulp tissue can either be capped directly or partially removed. In such cases, local areas of severe inflammation and micro-abscesses can be present coronally, whilst the apical parts of the pulp remain healthy [6]. From a histological perspective, it is conceivable that better healing should be achieved after partial removal of the infected pulp tissue [6]. Currently used pulp capping materials including mineral trioxide aggregate (MTA), other hydraulic calcium silicate cements, and calcium hydroxide all have antimicrobial properties due to the high pH environment they induce when in contact with tissue fluids [7]. However, if placed on highly inflamed or necrotic and infected pulp tissue, these high-pH materials can also induce further necrosis rather than healing [8]. Higher success rates have been shown for the capping of accidental pulp exposures compared to carious pulp exposures, the two differing mainly in their extent of bacterial penetration into the pulp [9,10]. Unfortunately, neither clinical signs and symptoms nor results of the currently available diagnostic tests correlate to the histological state of the exposed pulp [11]. This is the main conundrum in the clinical management of exposed pulps after caries excavation.

In this context, one topic appears to not have been addressed in a systematic manner: the effect of soft tissue lavage after pulp exposure. Lavage of the wound was identified as the central means of disinfection for open and potentially infected wounds during the Great War [12]. Sodium hypochlorite (NaOCl) in aqueous solution was identified at that time as the agent of choice to chemo-mechanically clean such injuries. This was because of its unique and somewhat selective effect on necrotized and infected soft tissues [12,13], which also led to its use in the chemo-mechanical debridement of infected root canal systems [14]. Today, sodium hypochlorite solutions are the first choice in pulpectomy procedures, i.e., in full root canal treatments [15]. However, in the context of potentially necrotized and infected pulp wounds encountered during VPT procedures, it is unclear whether NaOCl solutions are of any benefit or even in routine use. In theory, the use of NaOCl could be highly beneficial for this purpose, as it decontaminates the pulp wound chemically and thus, in theory, allows the capping material to exert its effect on healthier tissue. Its use has also been encouraged in recent guidelines [3], yet the scientific basis for this recommendation appears to be elusive. High success rates have been shown for VPT after removal of bacterial contamination using NaOCl in both primary and permanent molars [16,17]. Based on these and other findings, it can be concluded that the step of disinfecting the pulp wound by means of a wound lavage and removing microbially infiltrated, necrotic pulp tissue before it is capped may have an impact on survival of the remaining pulp after VPT [18,19].

This qualitative systematic review aimed to identify the types of pulp wound lavage and their influence on the vital pulp therapy of permanent human teeth with carious pulp exposure in all prospective controlled and randomized controlled clinical trials on vital pulp therapy. Moreover, factors that may be associated with the use of sodium hypochlorite in this context were analyzed.

## 2. Materials and Methods

This systematic review was conducted according to a protocol registered at the International Prospective Register of Systematic Reviews. The reporting follows the Preferred Reporting Items for Systematic Reviews and Meta-analyses (PRISMA) guidelines for systematic reviews [20] (Table S1). The reviewed question was: “what is the influence of topical disinfectants on pulp survival in the vital pulp therapy of permanent teeth with a carious pulp exposure?” Based on the PICO (population, intervention, comparison, outcome) format the search was carried out for clinical trials on permanent human teeth with a carious pulp exposure (P) that received vital pulp therapy (I), compared different treatment protocols (C) and reported the pulp survival after intervention with a minimal recall interval of 6 months (O).

### 2.1. Data Sources and Literature Search

A systematic search was conducted in six life science databases (MEDLINE, Biosis, Cochrane, EMBASE, Scopus and Web of Science) in October 2019. The search plan included a combination of keywords and indexing vocabulary (MeSH terms). Table S2 is an example of the search used in MEDLINE. No language restrictions were applied. To ensure the accuracy of the search one reviewer (A.M.) manually screened relevant journals in the field for potentially relevant publications.

### 2.2. Study Selection and Inclusion Criteria

The titles and abstracts were screened by two independent reviewers (A.M., D.-K.R.) according to pre-defined inclusion and exclusion criteria. Studies were included if they:

(a) Were carried out on human permanent teeth with a carious pulp exposure that received a type of VPT (direct pulp capping, partial pulpotomy or full/pulp chamber pulpotomy);
(b) Used a prospective randomized or non-randomized clinical trial design including a test and control group [21];
(c) Reported the pulp survival after a recall duration of at least six months after intervention.

Excluded were all studies:

(a) On animals;
(b) On deciduous teeth;
(c) On teeth without pulp exposure (e.g., indirect pulp capping);
(d) On teeth with experimental, mechanical or traumatic pulp exposures;
(e) That were retrospective, such as case reports or case series without a control group;
(f) That were histological without clinical assessment of the outcome.

All the articles selected by either reviewer were chosen for the full text evaluation which again was carried out by the two reviewers independently. In case of disagreement, consensus was reached by discussion and third-party-arbitration (M.Z.).

### 2.3. Data-Extraction and Quality Assessment

Data was extracted and managed on electronic files which were piloted and adapted before the final assessment of all the selected studies. Details regarding study characteristics were noted such as year of publication, randomized and non-randomized factors, type of environment in which the clinical trials were carried out (single-center, multi-center), number of operators and type of VPT performed (direct pulp capping, partial pulpotomy, full/pulp chamber pulpotomy). Furthermore, information about the subjects was also listed including age of the subjects (mean, median or age range), sample size (*N* teeth per group), type of teeth (molars, premolars, incisors), stage of root development (mature, immature) and inclusion of spontaneously painful teeth (yes, no). Data regarding wound lavage was extracted in detail, with a focus on the type of irrigant used (NaOCl, saline etc.), application of cotton pellets (yes, no) and the time required to achieve hemostasis (min) if mentioned. Other intervention related factors were also considered such as the use of rubber dam and sterile instruments, type of capping material (hydraulic calcium silicate cements, calcium hydroxide, etc.), final restoration placed immediately after pulp capping or re-entry. In addition, outcome related factors such as recall intervals (months), rate of drop out (percentage) and pulp survival (percentage) at least six months after the intervention were collected. The relevant information was collected and used for further analysis.

All the identified trials were analyzed regarding the method used for pulp wound lavage. The clinical trials targeting the effect of pulp wound lavage on the outcome of VPT were also examined separately.

The quality of the studies was assessed using RoB-2 and ROBINS-I tools as recommended by the Cochrane Collaboration (methods.cochrane.org [22]). RoB-2 was used for RCTs and assesses bias in the domains of randomization, assignment and adhering to intervention, missing data, outcome measurement and selection of the reported result. ROBINS-I is the recommended tool for bias assessment of non-randomized clinical trials in the domains confounding, selection of participants, misclassification of interventions, deviation from the intended intervention, missing outcome data, measurement of the outcome and selection of the reported results.

### 2.4. Data Analysis and Statistic Evaluation

A descriptive data analysis was carried out and statistics was applied only where appropriate (JMP 10.0.0, SAS Institute, Cary, NC, USA). Cohen’s kappa coefficient was calculated to measure inter-rater reliability. Interaction between the use of NaOCl for pulp lavage and year of publication was assessed using Student’s t test. The correlation between the use of calcium silicate cement in one of the experimental groups and NaOCl for pulp wound lavage was analyzed using Fisher’s exact test. The alpha-type error was set at 5% (*p* < 0.05).

## 3. Results

**Figure 1:**
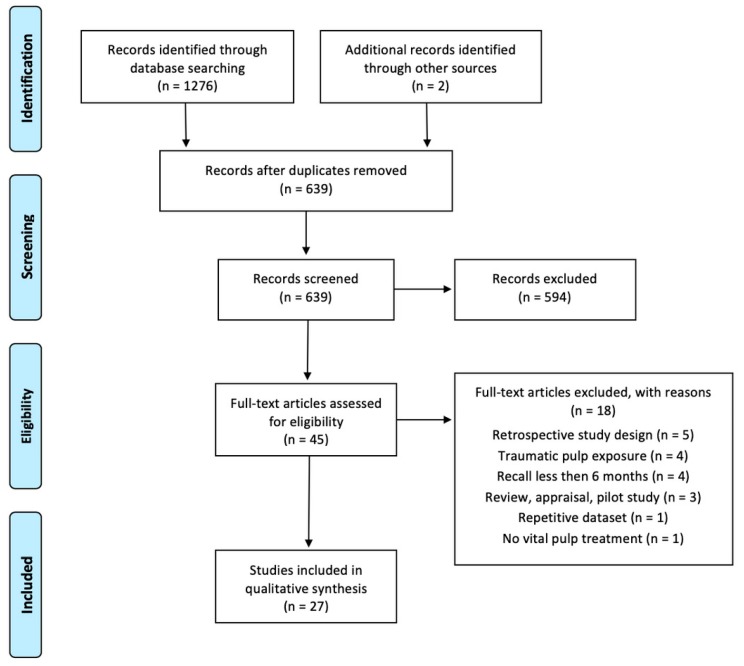
Flowchart of the data search that was carried out for the current investigation [20].

The systematic search initially identified 639 potentially eligible articles after duplicate removal (Figure 1). After screening of the titles and abstracts, 45 articles were selected and assessed in full-text. Eighteen out of these 45 articles were excluded at this stage (Table S3) and the other 27 selected for the qualitative synthesis [17,23,24,25,26,27,28,29,30,31,32,33,34,35,36,37,38,39,40,41,42,43,44,45,46,47,48]. Twenty-six of the selected articles were randomized clinical trials, one was non-randomized [47]. One publication [48] was in Chinese and was translated by a native speaker. There was almost perfect agreement between the two reviewers regarding full text evaluation (Cohen’s kappa = 0.91).

Although all the included studies showed a low to moderate risk of bias (Table S4), a large heterogeneity was found among them (Table 1). Because of this it was not possible to analyze the outcomes by means of a meta-analysis. Most of the identified trials examined the influence of the capping material (23/27) on pulp vitality in VPT [17,23,25,27,28,29,30,32,33,34,35,36,37,38,39,40,41,42,43,44,45,46,48], one of them also randomized the method used for pulp wound lavage [41]. The use of NaOCl solutions for pulp wound lavage was reported in 11 of the 27 included trials [17,25,31,33,34,36,39,41,42,45,46]. However, it was assessed as the randomized factor in just one of these 11 trials [41]. In ten trials the use of saline or water was reported [23,26,27,28,29,30,35,38,40,43]. Three trials mentioned a rinse with NaOCl only for some cases and the data was not stratified according to the type of irrigant used [24,32,37]. Anesthetic without vasoconstrictor was used to control bleeding from the pulp wound in one trial [44] and in another the pulp wound was disinfected with a cotton pellet containing 75% ethanol [48].

A clinical trial assessed the effect of glutaraldehyde in VPT in a non-randomized manner [47]. All the trials were carried out using conventional rotating instruments, except one in which an Erbium laser was also used in combination with saline lavage [28].

Most studies (16/27) reported a high percentage (>80%) of pulp survival after a recall duration of at least six months [17,23,24,25,27,29,31,32,33,36,37,40,41,42,43,47].

**Figure 2:**
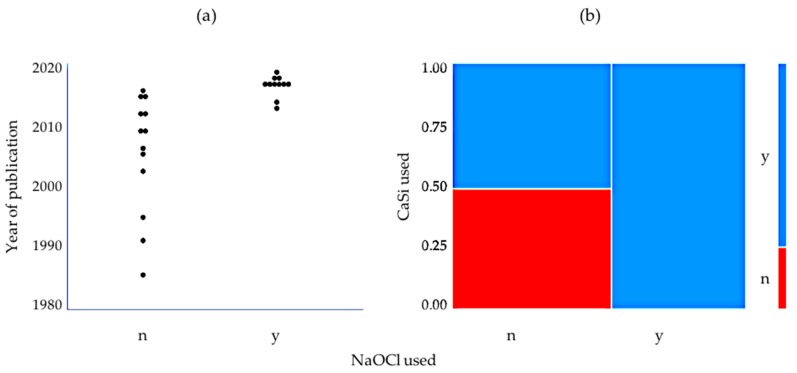
(**a**) Dot plot showing the use of an NaOCl solution in the trials included in this study according to the year of publication. There was a highly significant difference between the mean years of publication (Student’s *t*-test, *p* < 0.005). (**b**) Mosaic plot depicting the use of NaOCl for pulp lavage according to the use of calcium silicate cement (CaSi) for pulp capping in the trial (Fisher’s exact test, *p* < 0.05).

A statistical analysis was carried out to identify possible factors that may be correlated to the use of an NaOCl solution for pulp wound lavage. For this purpose, the three trials that used NaOCl in selected cases only but did not stratify the data were excluded [24,32,37]. A highly significant (*p* < 0.005) tendency was found for authors to use NaOCl for chemical soft tissue debridement in the more recent studies (Figure 2a). Moreover, the use of NaOCl was significantly (*p* < 0.05) correlated to the application of a hydraulic calcium silicate cement in the trials (Figure 2b).

Two of the included 27 trials explicitly compared different methods of pulp wound lavage [41,47]. Ozgur et al. (2017) randomized the use of 2.5% NaOCl to saline for wound lavage in partial pulpotomy. This study was carried out on 80 asymptomatic, immature molars. The subjects were between 6 and 13 y of age and were treated by one operator. Besides randomizing the pulp wound lavage with NaOCl or saline, two different capping materials-calcium hydroxide or MTA were also randomized in this trial. Hence, in total, there were four groups of 20 teeth each. The reported pulp survivals after one year were between 94% and 100% with no significant differences between the groups. No significant influence of disinfection with NaOCl could be shown and the results for both capping materials were comparable. A calculation of sample size was not reported.

An older clinical trial examined the effect of the use of glutaraldehyde in coronal pulpotomy [47]. This clinical trial was carried out on 20 immature, asymptomatic molars of 8–9 y old patients, divided into two groups. After bleeding was controlled with a wet cotton pellet, in one group a cotton pellet soaked with 5% buffered glutaraldehyde solution was placed on the amputated pulp for 5 min and then pulp capping was carried out using a calcium hydroxide paste mixed with 5% glutaraldehyde. In the other group, the calcium hydroxide paste was mixed with water and no additional chemical was used on the pulp wound before that. This trial had a recall rate of 100% after one year and reported pulp survival rates of 100% for both groups. However, it was carried out on a relatively small sample of only 10 teeth per group.

## 4. Discussion

This search revealed two studies that specifically assessed the effect of chemical agents used for pulp wound lavage on the pulp survival after VPT of carious teeth. Unfortunately, both studies were underpowered and no conclusions can be drawn based on this data. Despite a lack of evidence, there appears to be a trend towards using NaOCl to clean pulp wounds in the VPT of teeth with carious exposures.

The topic under investigation has not been given the attention it probably deserves. Since it is not possible to precisely identify the level of pulp infection in carious exposures by means of the presently available diagnostic tools, wound disinfection and debridement could be a core topic. Obviously, it is not. A recent review suggested that mechanically removing more pulp tissue in these situations can improve the rate of pulp survival, i.e., pulpotomy seems to yield better results than mere pulp capping [49]. From a histological point of view this makes sense, as the infection advances from the crown towards the root [50]. However, this statement needs to be weighed against the studies by Bjørndal [5] and Asgari and co-workers [24], which until now are the only trials that randomized these levels of intervention (Table 1). Neither found a difference between direct pulp capping and partial or full pulpotomy. Moreover, if more pulp tissue is removed mechanically, it will become harder to check the tooth clinically. Despite the absence of disease, such a tooth may no longer respond to simple clinical monitoring tests, such as the cold test [51]. Therefore, chemical wound debridement via lavage of the pulp wound using an antiseptic irrigant in conjunction with minimally invasive mechanical intervention could be the better option. Sodium hypochlorite solutions appear to be ideal for that purpose because of their unique effect on necrotic soft tissue [52] as well as biofilm [53]. Introduced more than 100 years ago, NaOCl solutions remain a standard of care in the disinfection of external soft tissue wounds [54]. Irrigants containing one or multiple antibiotics have also been discussed, as they selectively kill the invading microorganisms and obviously cause less collateral damage than non-specific biocides such as NaOCl [55]. There is also a history of glutaraldehyde usage in antiseptic solutions [56] and despite the fact that it is still contained in some dentine bonding agents [57], the high toxicity and mutagenic potential of this chemical would preclude it from the application proposed by Waly (1995) [47].

Both trials on the effects of wound lavage on the outcome of pulpotomy, identified by this search, were under-powered. The first had four groups and *N* = 20 [41], the second had two groups and *N* = 10 [47]. Nowadays, to be accepted by any internationally renowned journal, trials should be registered and a sample size analysis needs to be provided. One of the authors (M.Z.) is part of a team of researchers that planned and are executing a clinical trial on the topic: wound lavage in direct pulp capping of carious exposures in adult teeth using 2.5% NaOCl versus 0.9% saline solution. This trial has been registered (CTRI/2019/01/017167). The power analysis revealed that, assuming a 25% difference in pulp survival after one year between the two treatments and a 25% dropout rate, 48 patients per group are necessary to detect a significant difference with a chance (power) of 80% at a type I error of 0.05.

As shown in this review, the heterogeneity of the underlying studies is such that comparisons between individual treatment factors and outcomes in any form of meta-analyses would be spurious (Table 1). Success rates in the trials included in this review differed largely for similar interventions e.g., partial pulpotomy using saline as an irrigant and calcium hydroxide as a capping material. The nested clinical trial by Bjørndal and co-workers [5] has been criticized by some clinicians for its reported low success rates after carious exposure of the pulp. It has been suggested that this could be because of the use of an “outdated” pulp capping agent, a calcium hydroxide liner. However, the difference between calcium silicate and calcium hydroxide based capping materials seems to be minor (Table 1). Therefore, other factors that may have had an impact on the outcome must also be considered, such as clinical skills of the operators, inclusion of painful teeth in the trial sample, and/or the lack of disinfection of the pulp wound with NaOCl before capping. In spite or maybe also because of these possible procedural shortcomings, the Bjørndal trial set a new standard in terms of its high methodological quality and may have spurred more research. More recent trials showed better outcomes even when calcium hydroxide was used as the pulp capping material (Table 1) but they also used NaOCl for pulp lavage. Whether the use of NaOCl directly influenced the outcome of these trials, remains unclear and cannot be elucidated based on the presently available literature.

Although a direct comparison of the selected studies and assessing the influence of individual factors on the outcome of VPT was not possible, correlations between the use of NaOCl and other study parameters could still be identified. Our findings suggest that there is an increasing trend towards using NaOCl solutions for pulp wound lavage in the VPT of carious exposures despite the lack of scientific evidence for it. NaOCl solutions are a popular choice among endodontists [58] and many of the more recent trials identified by this search involved endodontists. This may also be why the recent consensus statement in a prominent endodontology journal recommends the use of NaOCl for pulp wound lavage, as most of the authors of this publication specialize in the field of endodontology [3]. However, practitioners of other specialties e.g., restorative dentistry, who also perform VPT clinically, may not necessarily be inclined to follow this protocol in the absence of available scientific evidence. Lastly, it is important to remember that it is not only the state of pulp tissue at the site of capping, but also the bacterial seal provided by subsequent restoration of the tooth that will determine the long-term success of VPT [59].

In view of the lack of scientific evidence in this context, future studies should assess different wound lavage concepts used in other fields of medicine and evaluate these regarding their effectiveness in VPT.

## 5. Conclusions

This systematic review identified a clear trend towards using sodium hypochlorite for pulp wound lavage during vital pulp therapy in recent trials and in combination with the use of calcium silicate cements as pulp capping materials. However, the effect of sodium hypochlorite for this purpose still needs to be substantiated by well-designed clinical trials.

## Figures and Tables

**Table 1:** Summary of the included studies.

Author/Year	Level of Bias ^1^	Level of Intervention ^2^	Randomized/Non-Randomized Factor	Number of Operators/Centers ^3^	*N* Groups ^4^	Immature Roots ^5^	Teeth Causing Pain ^6^	Irrigant Used for Wound Lavage ^7^	Capping Material ^8^	Rate of Pulp Survival ^9^
Asgary S. et al. 2013 [23]	low	FP	capping material	23 M	205–208 *2*	b	y	saline	CaSi	high
Asgary S. et al. 2018 [24]	some concerns	FP/PP/DPC	level of intervention	several S	69–76 *3*	n	n	0.2% CHX (5.25% NaOCl)	CaSi	high
Awawdeh L. et al. 2018 [25]	some concerns	DPC/FP	capping material	Ns S	34 *2*	n	y	5% NaOCl	CaSi	high
Björndal L. et al. 2010 [26]	low	DPC/PP	level of intervention	several M	27–31 *2*	n	y	saline	Ca(OH)_2_ liner	low
Brizuela C. et al. 2017 [27]	low	DPC	capping material	5 S	53–60 *3*	b	ns	saline	Ca(OH)_2_/CaSi	high
Cengiz E. et al. 2016 [28]	some concerns	DPC	capping material/laser debridement	1 S	15 *4*	n	n	saline	Ca(OH)_2_/resin based CaSi	medium/high
Chailertvanitkul P. et al. 2014 [17]	low	PP	capping material	2 S	40–44 *2*	y	n	2.5% NaOCl	CaSi/Ca(OH)_2_ liner	high
El-Meligy O.A. et al. 2006 * [29]	low	FP	capping material	1 S	*15* *sm*	y	n	saline	Ca(OH)_2_/CaSi	high
Fitzgerald M. et al. 1991 [30]	some concerns	DPC	capping material	ns S	16–20 *2*	n	n	water	Ca(OH)_2_ liner	medium
Galani M. et al. 2017 [31]	low	FP	level of intervention	1 S	27 *2*	b	y	2.5% NaOCl	CaSi	high
Ghoddusi J. et al. 2012 [32]	some concerns	FP	capping material	ns S	13–15 *2*	y	n	saline or 2.5% NaOCl	CaSi/ZOE	high
Hedge S. et al. 2017 [33]	some concerns	DPC	capping material	1 S	12 *2*	n	n	3% NaOCl	CaSi	high
Hilton T.J. et al. 2013 [34]	some concerns	DPC	capping material	35 M	175–183 *2*	b	n	5.25% NaOCl	CaSi/Ca(OH)_2_	high/medium
Hodosh M. et al. 2003 [35]	low	DPC	capping material	ns S	12–18 *3*	n	n	water	PCA	low/medium/high
Kang C.M. et al. 2017 [36]	low	PP	capping material	several S	33–36 *3*	b	n	3% NaOCl	CaSi	high
Katge F.A. et al. 2017 * [37]	low	DPC	capping material	1 S	*29* *sm*	y	n	saline (3% NaOCl)	CaSi	high
Kumar V. et al. 2016 [38]	low	FP	capping material	1 S	17–19 *3*	n	y	saline	Ca(OH)_2_/CaSi/PRF	low/medium
Kundzina R. et al. 2017 [39]	low	DPC	capping material	6 M	33–37 *2*	n	y	0.5% NaOCl	Ca(OH)_2_ liner/CaSi	medium/high
Nosrat A. et al. 2013 [40]	low	FP	capping material	1 S	25–26 *2*	y	y	saline	CaSi	high
Ozgur B. et al. 2017 [41]	low	PP	wound lavage/capping material	1 S	20 *4*	y	n	2.5% NaOCl or saline	CaSi/Ca(OH)_2_	high
Parinyaprom N. et al. 2018 [42]	low	DPC	capping material	8 S	29–30 *2*	b	ns	2.5% NaOCl	CaSi	high
Qudeimat M.A. et al. 2007 [43]	low	PP	capping material	ns	32 *2*	b	n	saline	CaSi/Ca(OH)_2_	high
Santini A.H. et al. 1985 * [44]	low	FP	capping material	ns	*130* *sm*	b	y	local anaesthetic	Ca(OH)_2_ liner without/with LM	medium/low
Suhag K. et al. 2019 [45]	low	DPC	capping material	1 S	32 *2*	n	n	2.5% NaOCl	Ca(OH)_2_/CaSi	medium/high
Taha N.A. et al. 2017 [46]	low	PP	capping material	ns S	23–27 *2*	n	y	2.5% NaOCl	Ca(OH)_2_/CaSi	medium/high
Waly N.G. 1995 [47]	low	FP	wound lavage	ns S	10 *2*	y	n	glutaraldehyde or water	Ca(OH)_2_	high
Wei et al. 2010 [48]	some concerns	DPC	capping material	ns S	54–59 *2*	b	n	75% ethanol	Ca(OH)_2_ liner/NH	medium

^1^ A summary of the risk of bias analysis can be reviewed in Table S4. ^2^ FP: full pulpotomy; PP: partial pulpotomy; DPC: direct pulp capping. ^3^ S: single center; M: multicenter; ns: not specified, several: number of operators not specified but mentioned that there were several. ^4^ N: teeth per group, groups: number of groups analyzed in the study, (sm) split mouth design; (some studies also had treatment groups with indirect pulp capping or root canal treatments, that were not considered for this study). ^5^ y: only immature teeth; b: both mature and immature teeth; n: no immature teeth/only teeth with complete root formation included or studies that did not explicitly state the stage of root development, but were carried out only on adult patients (above 18 years of age). ^6^ y: yes, teeth causing spontaneous pain were included in these trials; n: no; ns: not specified. ^7^ NaOCl: sodium hypochlorite; (in brackets) second irrigant which was only used for selected cases; italic: if the irrigant was the randomized factor; in one trial, Ghoddusi et al. 2012 either saline or NaOCl was used and it was not stated which was used when. ^8^ CaSi: calcium silicate cement; Ca(OH)_2_: calcium hydroxide, Ca(OH)_2_ liner: calcium hydroxide liner; ZOE: zinc-oxide eugenol; PCA: polycarboxylate cement; PRF: platelet rich fibrin, LM: Ledermix^®^ (a paste containing tetracycline and corticosteroid), NH: nanohydroxyapatite. ^9^ Reported rate of pulp survival 6–12 months after intervention: low: less than 40%; medium: 40–80%; high: more than 80%; some studies reported varying success rates for the different treatment groups. * These studies had a split mouth design; all the studies listed above were randomized clinical trials except Waly 1995 which was a non-randomized clinical trial and Awawdeh et al. 2018 which was pseudorandomized (a coin was tossed and the next tooth assigned to the other group). Note: not all of the collected data is presented in this communication, but is available upon request from the corresponding author.

## Supplementary Materials

The following are available online at https://www.mdpi.com/2077-0383/9/4/984/s1, Table S1: Prisma checklist, Table S2: Example of search strategy, Table S3: Studies excluded after full text evaluation, Table S4: Bias assessment.
