# Supplementary material for: Wound Lavage in Studies on Vital Pulp Therapy of Permanent Teeth with Carious Exposures: A Qualitative Systematic Review
Source: J Clin Med. 2020 Apr 1;9(4):984. doi: 10.3390/jcm9040984 (PMC7231275; doi:10.3390/jcm9040984)
Supplement: Supplementary file 1 [file jcm-09-00984-s001.zip › jcm-746209-SI/Supplement material/Table S2.docx]

**Table 2.** Example of search strategy (here in Medline database/OVID) employed for this literature review.

| **Number** | **Search history** | **Results** |
| --- | --- | --- |
| 1 | ((Pulpotomy/ or Dental Pulp Capping/ or (pulpotomy or pulp-capping or ("pulp cap" adj3 (procedure or method or technique))).ti,ab.) and (exp dental caries/ or exp dental fissures/ or (caries or carious).ti,ab. or Dental Pulp Exposure/ or (pulp adj3 expos*).ti,ab.)) not exp Tooth, Deciduous/ | 986 |
| 2 | "Pulp Capping and Pulpectomy Agents"/ or Calcium Hydroxide/ or ("calcium hydroxide" or calasept or calxyl or hypocal or limewater or pulpdent or "mineral trioxide aggregate" or mineral-trioxide-aggregate or MTA or "hydraulic cements" or biodentine or ((adhesive or bonding) adj3 composite) or cariosolv or formocresol or (capping adj3 (material* or method* or technique* or agent* or disinfectant*))).ti,ab. | 13746 |
| 3 | 1 and 2 | 618 |
| 4 | 3 not (exp In Vitro Techniques/ or "in vitro".ti.) | 598 |
| 5 | (RANDOMIZED CONTROLLED TRIAL/ or CONTROLLED CLINICAL TRIAL/ or RANDOM ALLOCATION/ or DOUBLE BLIND METHOD/ or SINGLE BLIND METHOD/ or exp clinical trial/ or PLACEBOS/ or RESEARCH DESIGN/ or COMPARATIVE STUDY/ or exp EVALUATION STUDIES/ or FOLLOW UP STUDIES/ or PROSPECTIVE STUDIES/ or (clin$ adj25 trial$).ti,ab. or ((singl$ or doubl$ or trebl$ or tripl$) adj25 (blind$ or mask$)).ti,ab. or (placebo$ or random$ or crossover* or "cross over" or assign* or allocate* or crossingover* or factorial*).ti,ab. or (control$ or prospectiv$ or retrospectiv$ or follow-up or volunteer$).ti,ab.) not (ANIMALS not HUMANS).sh. | 6751148 |
| 6 | 4 and 5 | 253 |
