# Supplementary material for: Wound Lavage in Studies on Vital Pulp Therapy of Permanent Teeth with Carious Exposures: A Qualitative Systematic Review
Source: J Clin Med. 2020 Apr 1;9(4):984. doi: 10.3390/jcm9040984 (PMC7231275; doi:10.3390/jcm9040984)
Supplement: Supplementary file 1 [file jcm-09-00984-s001.zip › jcm-746209-SI/Supplement material/Table S3.docx]

**Table 3.** Studies excluded after full text evaluation.

| ***Reviews, appraisals or pilot studies (n = 3)*** |
| --- |
| [Abiraamasri and Deepa; 2018], [Browning; 2015], [Yazdanfar et al.; 2015] |
|  |
| ***Retrospective studies (n = 5)*** |
| [Caliskan and Guneri; 2017], [Cho et al.; 2013], [Horsted et al.; 1985], [Mente et al.; 2010],  [Mente et al.; 2014] |
|  |
| ***Recall duration of less than 6 months (n = 4)*** |
| [Arafa et al.; 2019], [Eftimoska et al.; 2015], [Lee et al.; 2015], [Song et al.; 2015] |
|  |
| ***Data collected on teeth with traumatic or mechanical pulp exposures or data pooled for teeth with both carious and traumatic pulp exposures (n = 4)*** |
| [Dabrowska et al.; 1997] [Jang et al.; 2015], [Moritz et al.; 1998], [Olivi et al.; 2007] |
|  |
|  |
| ***No pulp exposure, indirect pulp capping (n = 1)*** |
| [Vural et al.; 2017] |
|  |
|  |
| ***Study carried out on the same data set as an already included study (n = 1)*** |
| [Bjorndal et al.; 2017] |

**References**

1. Abiraamasri, B. L.; Deepa, D. G. Comparison of ferric sulphate and calcium hydroxide as a pulpotomy agent. *Research Journal of Pharmacy and Technology* **2018**, *11*, 1881-1883.

2. Browning, W. D. 2015 Update: Approaches to Caries Removal. *Journal of Esthetic and Restorative Dentistry* **2015**, *27*, 383-385.

3. Yazdanfar, I.; Gutknecht, N.; Franzen, R. Effects of diode laser on direct pulp capping treatment: A pilot study. *Lasers in Medical Science* **2015**, *30*, 1237-1243.

4. Caliskan, M. K.; Guneri, P. Prognostic factors in direct pulp capping with mineral trioxide aggregate or calcium hydroxide: 2- to 6-year follow-up. *Clinical Oral Investigations* **2017**, *21*, 357-367.

5. Cho, S. Y.; Seo, D. G.; Lee, S. J.; Lee, J.; Lee, S. J.; Jung, I. Y. Prognostic factors for clinical outcomes according to time after direct pulp capping. *Journal of Endodontics* **2013**, *39*, 327-331.

6. Horsted, P.; Sandergaard, B.; Thylstrup, A.; El Attar, K.; Fejerskov, O. A retrospective study of direct pulp capping with calcium hydroxide compounds. *Endodontics & Dental Traumatology* **1985**, *1*, 29-34.

7. Mente, J.; Geletneky, B.; Ohle, M.; Koch, M. J.; Friedrich Ding, P. G.; Wolff, D.; Dreyhaupt, J.; Martin, N.; Staehle, H. J.; Pfefferle, T. Mineral trioxide aggregate or calcium hydroxide direct pulp capping: an analysis of the clinical treatment outcome. *Journal of Endodontics* **2010**, *36*, 806-813.

8. Mente, J.; Hufnagel, S.; Leo, M.; Michel, A.; Gehrig, H.; Panagidis, D.; Saure, D.; Pfefferle, T. Treatment outcome of mineral trioxide aggregate or calcium hydroxide direct pulp capping: long-term results. *Journal of Endodontics* **2014**, *40*, 1746-1751.

9. Arafa, A.; Kenawi, L. M. M.; Issa, N. Assessment of reparative hard tissue formation after direct pulp capping with Biodentine versus mineral trioxide aggregate. *Endo-Endodontic Practice Today* **2019**, *13*, 227-236.

10. Eftimoska, M.; Apostolska, S.; Rendzhova, V.; Gjorgievska, E.; Stevanovic, M.; Ivanovski, K.; Jankulovska, M.; Elenchevski, S.; Pavlevska, M.; Dimkov, A. Clinical and histological analyzes of the response of the pulp after its direct capping with Calxyl, MTA and Biodentine. *Research Journal of Pharmaceutical, Biological and Chemical Sciences* **2015**, *6*, 1097-1111.

11. Lee, L. W.; Hsiao, S. H.; Hung, W. C.; Lin, Y. H.; Chen, P. Y.; Chiang, C. P. Clinical outcomes for teeth treated with electrospun poly(epsilon-caprolactone) fiber meshes/mineral trioxide aggregate direct pulp capping. *Journal of Endodontics* **2015**, *41*, 628-636.

12. Song, M.; Kang, M.; Kim, H. C.; Kim, E. A randomized controlled study of the use of ProRoot mineral trioxide aggregate and Endocem as direct pulp capping materials. *Journal of Endodontics* **2015**, *41*, 11-15.

13. Dabrowska, E.; Zdanowicz-Wiloch, J.; Pawinska-Magnuszewska, M.; Stokowska, W. Intravital treatment of the pulp with simultaneous laser biostimulation. *Roczniki Akademii Medycznej W Bialymstoku* **1997**, *42*, 168-176.

14. Jang, Y.; Song, M.; Yoo, I. S.; Song, Y.; Roh, B. D.; Kim, E. A Randomized Controlled Study of the Use of ProRoot Mineral Trioxide Aggregate and Endocem as Direct Pulp Capping Materials: 3-month versus 1-year Outcomes. *Journal of Endodontics* **2015**, *41*, 1201-1206.

15. Moritz, A.; Schoop, U.; Goharkhay, K.; Sperr, W. The CO2 laser as an aid in direct pulp capping. *Journal of Endodontics* **1998**, *24*, 248-251.

16. Olivi, G.; Genovese, M. D.; Maturo, P.; Docimo, R. Pulp capping: advantages of using laser technology. *European Journal of Paediatric Dentistry* **2007**, *8*, 89-95.

17. Vural, U. K.; Kiremitci, A.; Gokalp, S. Clinical assessment of mineral trioxide aggregate in the treatment of deep carious lesions. *Nigerian Journal of Clinical Practice* **2017**, *20*, 600-604.

18. Bjorndal, L.; Fransson, H.; Bruun, G.; Markvart, M.; Kjaeldgaard, M.; Nasman, P.; Hedenbjork-Lager, A.; Dige, I.; Thordrup, M. Randomized Clinical Trials on Deep Carious Lesions: 5-Year Follow-up. *Journal of Dental Research* **2017**, *96*, 747-753.
