# Supplementary material for: Wound Lavage in Studies on Vital Pulp Therapy of Permanent Teeth with Carious Exposures: A Qualitative Systematic Review
Source: J Clin Med. 2020 Apr 1;9(4):984. doi: 10.3390/jcm9040984 (PMC7231275; doi:10.3390/jcm9040984)
Supplement: Supplementary file 1 [file jcm-09-00984-s001.zip › jcm-746209-SI/Supplement material/Table S4.docx]

**Table 4.** Risk of bias assessment with RoB 2 and ROBINS-I. RoB 2 risk of bias assessment for randomized clinical trials

| **Study** | **Randomization bias** | **Effect of adhering to intervention** | **Missing data bias** | **Outcome measurement** | **Selection of the reported result** | **Overall** |
| --- | --- | --- | --- | --- | --- | --- |
| Asgary S. et. al. 2013 | 1.1 Y  1.2 PY  1.3 N  low | 2.1 PN  2.2 N  2.4 N  2.5 PN  low | 3.1 Y  low | 4.1 N  4.2 N  4.3 N  low | 5.1 Y  5.2 N  5.3 PN  low | low |
| Asgary S. et. al. 2018_1_ | 1.1 Y  1.2 PY  1.3 N  low | 2.1 N  2.2 Y  2.3 PN  2.4 PY  some concerns | 3.1 PN  3.2 PY  low | 4.1 N  4.2 N  4.3 PY  4.4 PN  low | 5.1 PY  5.2 N  5.3 PN  low | some concerns |
| Awawdeh L. et. al. 2018_2_ | 1.2 NI  1.3 N  some concerns | 2.1 N  2.2 PY  2.3 PY  2.4 PN  2.5 PN  low | 3.1 Y  low | 4.1 N  4.2 N  4.3 NI  4.4 PN  4.5 N  some concerns | 5.1 PY  5.2 N  5.3 PN  low | some concerns |
| Björndal L. et. al. 2010 | - 1. Y   2. PY   3. N   low | 2.1 PN  2.2 Y  2.3 PY  2.4 PN  2.5 PN  low | 3.1 Y  low | 4.1 N  4.2 PN  4.3 N  low | 5.1 PY  5.2 N  5.3 PN  low | low |
| Brizuela C. et. al. 2017^3^ | 1.1 Y  1.2 Y  1.3 N  low | 2.1 N  2.2 PY  2.3 PY  2.4 PN  2.5 PN  low | 3.1 N  3.2 PY  low | 4.1 N  4.2 PN  4.3 PY  4.4 PN  low | 5.1 PY  5.2 PN  5.3 PN  low | low |
| Cengiz E. et. al. 2016^4^ | 1.2 NI  1.3 NI  some concerns | 2.1 PY  2.2 Y  2.3 PY  2.4 PN  2.5 PN  low | 3.1 yes  low | 4.1 N  4.2 N  4.3 PN  low | 5.1 PY  5.2 PN  5.3 PN  low | some concerns |
| Chailertvanitkul P. et. al. 2014 | 1.1 Y  1.2 Y  1.3 PN  low | 2.1 N  2.2 PY  2.3 PY  2.4 PN  2.5 PN  low | 3.1 Y  low | 4.1 N  4.2 N  4.3 N  low | 5.1 PY  5.2 N  5.3 N  low | low |
| El-Meligy O.A. et. al. 2006 | 1.1 Y  1.2 PY  1.3 N  low | 2.1 PN 2.2 PY  2.3 PY  2.4 PN  2.5 PN  low | 3.1 Y  low | 4.1 N  4.2 N  4.3 N  low | 5.1 PY  5.2 N  5.3 PN  low | low |
| Fitzgerald M. et. al. 1991 | 1.2 NI  1.3 N  some concerns | 2.1 PN 2.2 PY  2.3 PY  2.4 PN  2.5 PN  low | 3.1 Y  low | 4.1 N  4.2 N  4.3 N  low | 5.1 PY  5.2 N  5.3 PN  low | some concerns |
| Galani M. et. al. 2017 | 1.1 Y  1.2 PY  1.3 N  low | 2.1 PY  2.2 PY  2.3 PY  2.4 N  2.5 N  low | 3.1 Y  low | 4.1 N  4.2 PN  4.3 N  low | 5.1 PY  5.2 PN  5.3 PN  low | low |
| Ghoddusi J. et. al. 2012^5^ | 1.2 NI  1.3 NI  some concerns | 2.1 PN  2.2 PY  2.3 N  2.4 PN  2.5 PN  low | 3.1 PY  low | 4.1 N  4.2 PN  4.3 NI  4.4 NI  4.5 PN  some concerns | 5.1 PY  5.2 PN  5.3 PN  low | some concerns |
| Hedge S. et. al. 2017^6^ | 1.1 Y  1.2 Y  1.3 N  low | 2.1 PN  2.2 PY  2.3 PY  2.4 N  2.5 N  low | 3.1 Y  low | 4.1 PN  4.2 PN  4.3 PY  4.4 NI  4.5 PN  some concerns | 5.1 PY  5.2 PN  5.3 PN  low | some concerns |
| Hilton T.J. et. al. 2013^7^ | 1.1 PY  1.2 PY  1.3 N  low | 2.1 PY  2.2 Y  2.3 NI  2.6 PY  some concerns | 3.1 PY  low | 4.1 PN  4.2 PN  4.3 PY  4.4 PY  4.5 PN  some concerns | 5.1 PY  5.2 N  5.3 N  low | some concerns |
| Hodosh M. et. al. 2003 | 1.1 Y  1.2 Y  1.3 N  low | 2.1 N  2.2 N  2.4 N  2.5 N  low | 3.1 Y  low | 4.1 N  4.2 PN  4.3 PN  low | 5.1 Y  5.2 PN  5.3 PN  low | low |
| Kang C.M. et. al. 2017 | 1.1 Y  1.2 PY  1.3 PN  low | 2.1 N  2.2 PY  2.3 PY  2.4 N  2.5 N  low | 3.1 Y  low | 4.1 N  4.2 N  4.3 N  low | 5.1 PY  5.2 N  5.3 N  low | low |
| Katge F.A. et. al. 2017 | 1.1 Y  1.2 Y  1.3 N  low | 2.1 PN  2.2 Y  2.3 PN  2.6 Y  low | 3.1 PY  low | 4.1 N  4.2 N  4.3 PY  4.4 PN  low | 5.1 Y  5.2 PN  5.3 PN  low | low |
| Kumar V. et. al. 2016 | 1.1 Y  1.2 Y  1.3 N  low | 2.1 PN  2.2 Y  2.3 PY  2.4 N  2.5 N  low | 3.1 Y  low | 4.1 N  4.2 PN  4.3 PN  low | 5.1 PY  5.2 PN  5.3 PN  low | low |
| Kundzina R. et. al. 2017 | 1.1 Y  1.2 PY  1.3 N  low | 2.1 N  2.2 N  2.3 PY  2.4 PN  2.5 N  low | 3.1 Y  low | 4.1 N  4.2 PN  4.3 PN  low | 5.1 Y  5.2 N  5.3 PN  low | low |
| Nosrat A. et. al. 2013 | 1.1 Y  1.2 Y  1.3 N  low | 2.1 PN  2.2 PY  2.3 PY  2.4 N  2.5 N  low | 3.1 Y  low | 4.1 N  4.2 PN  4.3 N  low | 5.1 PY  5.2 PN  5.3 PN  low | low |
| Özgür B. et. al. 2017 | 1.1 Y  1.2 Y  1.3 N  low | 2.1 N  2.2 Y  2.3 PY  2.4 PN  2.5 PN  low | 3.1 Y  low | 4.1 N  4.2 N  4.3 N  low | 5.1 Y  5.2 N  5.3 PN  low | low |
| Parinyaprom N. et. al. 2018^8^ | 1.1 Y  1.2 Y  1.3 N  low | 2.1 N  2.2 Y  2.3 PY  2.4 PN  2.5 PN  low | 3.1 Y  low | 4.1 N  4.2 N  4.3* PY  4.4 PN  low | 5.1 PY  5.2 PN  5.3 PN  low | low |
| Qudeimat M.A. et. al. 2007 | 1.1 Y  1.2 PY  1.3 N  low | 2.1 PN  2.2 PY  2.3 PY  2.4 N  2.5 N  low | 3.1 Y  low | 4.1 N  4.2 N  4.3* PY  4.4 PN  low | 5.1 PY  5.2 PN  5.3 PN  low | low |
| Santini A.H. et. al. 1985 | 1.1 Y  1.2 Y  1.3 N  low | 2.1 NI  2.2 Y  2.3 PY  2.4 PN  2.5 PN  low | 3.1 PN  3.2 N  3.3 PN  low | 4.1 N  4.2 PN  4.3 PY  4.4 PN  low | 5.1 PY  5.2 PN  5.3 PN  low | low |
| Suhag K. et. al. 2019 | 1.1 Y  1.2 Y  1.3 N  low | 2.1 N  2.2 Y  2.3 Y  2.4 PN  2.5 PN  low | 3.1 Y  low | 4.1 N  4.2 N  4.3 PN  low | 5.1 Y  5.2 PN  5.3 PN  low | low |
| Taha N.A. et. al. 2017 | 1.1 Y  1.2 Y  1.3 N  low | 2.1 PN  2.2 PY  2.3 PY  2.4 PN  2.5 PN  low | 3.1 Y  low | 4.1 N  4.2 N  4.3* PY  4.4 PN  low | 5.1 PY  5.2 PN  5.3 PN  low | low |
| Wei et.al.2010^9^ | 1.2 NI  1.3 N  some concerns | 2.1 NI  2.2 NI  2.3 NI  2.4 PN  2.5 PN  low | 3.1 Y  low | 4.1 N  4.2 PN  4.3 NI  4.4 NI  4.5 PN  some concerns | 5.1 PY  5.2 PN  5.3 PN  low | some concerns |

**ROBINS-I risk of bias assessment for controlled clinical trials.**

| **Study** | **Confounding** | **Selection of participants** | **Classification bias** | **Deviation from intended intervention** | **Missing data** | **Outcome measurement** | **Bias in the selection of the result** | **Overall** |
| --- | --- | --- | --- | --- | --- | --- | --- | --- |
| Waly N.G. 1995 | 1.1 N  low | 2.1 PN  2.4 PY  low | 3.1 Y  3.2 PY  3.3 PN  low | 4.1 N  4.3 PY  4.4 Y  4.5 Y  low | 5.1 Y  5.2 N  5.3 N  low | 6.1 PN  6.2 PY  6.3 Y  6.4 N  low | 7.1 N  7.2 PN  7.3 N  low | low |

Y= yes N = no PY = probably yes PN = probably no NI = no information provided.
